# A national retinoblastoma network: experiences in Kenya and the UK

**Published:** 2018-06-03

**Authors:** Ashwin Reddy, Kahaki Kimani

**Affiliations:** 1 Lead Clinician for Ophthalmology and Retinoblastoma Services: Royal London Hospital, London, UK.; 2 Senior Lecturer: University of Nairobi, Nairobi, Kenya.

**Retinoblastoma is a rare disease. Saving the lives of the children affected requires early detection, good referral systems and expert care by a multidisciplinary team. Setting up a national and joined-up service is an essential first step.**

**Figure:**
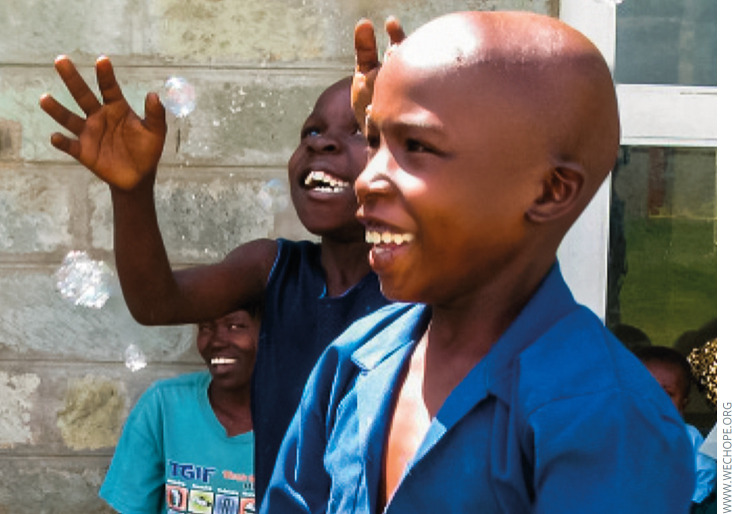
Children who are part of the Child Life programme for retinoblastoma play outside the national retinoblastoma centre in Kenyatta. KENYA

Because retinoblastoma is rare, it is sensible to gather those people with the expertise to manage children with retinoblastoma (and the resources and equipment to do so) in one or more designated national centres. In large countries, with high birth rates, several centres must be designated. All retinoblastoma centres in a country need to work together as part of a national service with standard management protocols. The national service must be audited on a regular basis and the data shared at national meetings where all centres that see and treat children with retinoblastoma are represented.

## Experiences in Kenya

A study in the year 2000 reviewed the presentation and management of retinoblastoma at Kenyatta National Hospital (the largest referral hospital in Kenya). It showed that there were significant gaps in the management of patients presenting with retinoblastoma. This led to poor outcomes for patients, many of whom were dying. In an attempt to bridge some of these gaps, a retinoblastoma working group was formed involving ophthalmologists, paediatric oncologists, radiation oncologists and pathologists. Due to limited funding and competing job demands, this working group had limited success.

In 2007, the group was contacted by Daisy's Eye Cancer fund (now known as World Eye Cancer Hope) who, together with a retinoblastoma expert from the Hospital for Sick Kids, Toronto, reignited the determination to improve the management of patients with retinoblastoma in Kenya. With their financial support and expertise, the first Kenya National Retinoblastoma meeting was held in 2008 and participants from all over Kenya attended, including ophthalmologists, paediatric oncologists, radio-oncologists, pathologists, ophthalmic clinical officers, nurses, parents of children with retinoblastoma, retinoblastoma survivors and a child-life specialist (see panel overleaf).

A situation analysis revealed that the challenges were similar in all regions of the country and included:

- Lack of awareness about the disease both among the public as well as medical workers
- Poor referral networks
- Lack of psychosocial and financial support for affected families
- Lack of standardised management protocols
- Delayed and often scanty histopathology reports
- Lack of chemotherapy drugs
- Lack of communication between referral centres
- Poor follow-up of patients.

To address these issues, the group set four broad objectives (with attendant activities).

### 1. Standardise the management of retinoblastoma

- Design a standardised treatment protocol
- Standardise histopathology reporting using standard request and reporting forms
- Prepare a chemotherapy regimen according to international standards
- Establish sources and funding for the chemotherapeutic agents.

### 2. Improve awareness about retinoblastoma

- Education of primary health care workers especially those in maternal child health
- Use of the media, retail chains, transport and communication industry to spread awareness among the public
- Nationwide posters about Rb.

### 3. Develop partnerships for resource mobilisation

- Target faith-based organisations, non-governmental organisations, corporates, community development fund and local government authorities for funding
- Organise fund raising events like marathons/walks.

### 4. Provide psychosocial support to patients and their families

- Establish an association / support group of affected families
- Create information resources including a website and pamphlets
- Construct accommodation facilities for families of children undergoing long term treatment, especially those from remote areas.

Four committees were set up, one for each objective, and given tasks to be accomplished each year. A steering committee was formed to coordinate annual meetings and oversee the activities of each committee.

The Kenya National Retinoblastoma Strategy (KNRbS) was launched and included everyone who attended the first meeting. An annual KNRbS meeting was held for the next six years, during which achievements of the previous year would be reviewed and new tasks set out.

In those six years, the KNRbS was able to achieve the following:

- Ratification, publication and distribution of Kenya Retinoblastoma Best Practice Guidelines
- Improved access to chemotherapy
- Improved globe salvage therapy
- Inclusion of eye inspection for white reflex and squint as part of routine maternal child health care
- Establishment of a fund to enable each family affected by retinoblastoma to enrol in the National Health Insurance Fund which covers almost all aspects of retinoblastoma treatment, as well as other diseases.

## Experiences in the UK

The development of national services in the UK occurred because of concerns that uncommon children's conditions with a risk of mortality were being treated in units that were under-equipped to treat them.

It is advisable to have two centres in the UK in case one centre is unable to treat the condition, say due to lack of staff or closure due to infection for example.

A true multidisciplinary team evolves over time. Core medical staff members should include ophthalmologists, paediatric oncologists and histopathologists. Ideally there should be two of each so that leave is covered and colleagues can give advice when difficult situations arise either informally or at multidisciplinary meetings.

**Figure 1:**
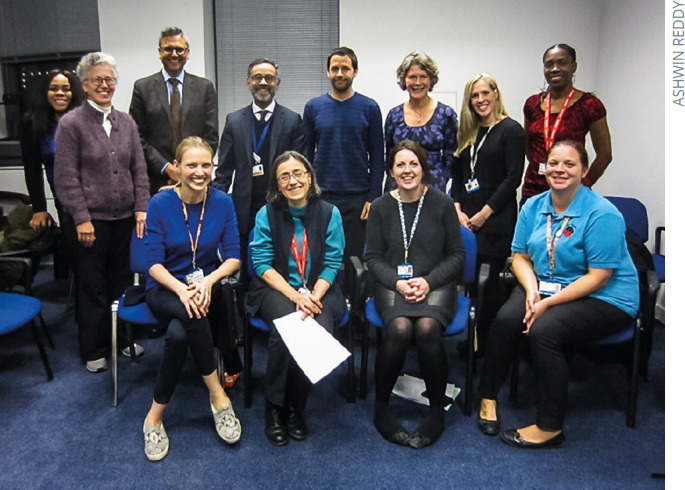
Core members of the Royal London Retinoblastoma Team, Barts Health NHS Trust, London, UK

Additional support staff are needed. In the UK, support staff include Clinical Nurse Specialists in oncology and ophthalmology, orthoptists to monitor vision in children, psychologists and a representative from the retinoblastoma patient support group in the UK (Childhood Eye Cancer Trust: CHECT). Figure 1.

Every support staff member has an important role to fulfil. The representative from CHECT is important as the parents feel that there is someone to speak to who is not a member of the hospital. Parents are likely to become distressed and this will affect the child and other members of the family. Counselling either by a psychologist or a member of staff (who has an understanding of the trauma that the family is undergoing) is of great benefit.

We hold multidisciplinary meetings with all staff members at least once a month to discuss patients who have had enucleations and treatment.

**Child Life: improving treatment and saving costs by caring for the whole child**

Retinoblastoma treatment is a stressful and potentially traumatic experience for children, who have to cope with the challenges of hospitalisation, illness, and disability. Child life specialists (known as play therapists or play specialists in some countries) work closely with children and families in these situations, offering emotional support practical tools and advice to help them cope better.

Child life specialists help to explain medical jargon to parents and children, and prepare them for procedures, usually through play.

**Figure 2:**
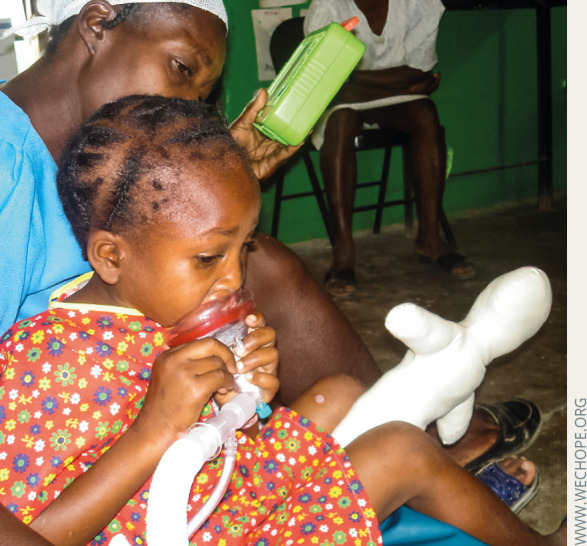
A child practises the use of a mask, Kenya

In Figure 2, a child is practising the use of a mask in preparation for surgery. She overcame her initial apprehension by first putting the mask on a simple cloth doll (a pattern that can be easily sewn by people in the local community for very little money).

The father also demonstrates one of the four key comfort positions (back to chest), which gives solid comfort to the child during this procedure. See **http://bit.ly/comfortpositions**.

These techniques reduce stress and can save costs: if a child is calm, and the parent knows what to do, a procedure can be completed by just one medical professional, rather than two or more.

